# Perceived social support and COVID-19 impact on quality of life in college students: an observational study

**DOI:** 10.1080/07853890.2022.2154943

**Published:** 2022-12-15

**Authors:** Ana Cahuas, Michele Wolf Marenus, Varun Kumaravel, Andy Murray, Kathryn Friedman, Haley Ottensoser, Weiyun Chen

**Affiliations:** a Department of Psychology, University of Michigan, Ann Arbor, MI, USA; b School of Kinesiology, University of Michigan at Ann Arbor, Ann Arbor, MI, USA

**Keywords:** Social support, quality of life, COVID-19, college students

## Abstract

**Background:**

The purposes of this study were to assess the current status of perceived social support and COVID-19 impact on quality of life, to investigate the association of perceived social support with the COVID-19 impact on quality of life, and to examine differences in perceived social support between better and worse COVID-19 impact on quality of life for the total sample and by gender.

**Methods:**

Participants included 1296 university students (399 male, 871 female, 22 transgender, non-binary, or other) with a mean age of 21.5 (SD  =  2.6 years) from a large public university in the Midwest region of the US. Students voluntarily completed two questionnaires and demographic information via Qualtrics based on a cross-sectional study design. The Multidimensional Scale of Perceived Social Support (MSPSS) is a 12-item survey used to assess an individual’s perception of social support from significant others, friends, and family. The COVID-19-Impact on Quality of Life scale (COVID-19 QoL) is a 6-item scale used to assess the impact of COVID-19 on quality of life. Data was analyzed using descriptive statistics, multiple linear regression, independent *t*-tests, and ANCOVA.

**Results:**

Multiple linear regression showed that perceived social support from family was a significant predictor of COVID-19 QoL (*F* = 35.154, *p* < .01) for the total sample. Further, *t-*test demonstrated significant differences between males and females on perceived social support (*t* = −2.184, *p* < .05) as well as COVID-19 QoL (*t* = −5.542, *p* < .01). Results of ANCOVA demonstrated a significant group effect on perceived social support for both males (*F* = 10.054, *p* < .01, *η^2^* = .025) and females (*F* = 5.978, *p* < .05, *η^2^* = 0.007), indicating that the better quality of life group scored higher on perceived social support than low quality of life.

**Conclusions:**

Social support from family may act as a key buffer for quality of life during the fall semester of 2020, amid the COVID-19 pandemic in college students. With social interactions restricted during COVID-19, maintained access to social support is highly important.

**KEY MESSAGES**
- Social support is a crucial contributing factor to the impact of COVID-19 on quality of life, and support from social relationships may buffer these challenging and unpredictable times.
- The COVID-19 pandemic may have impacted the quality of life of males and females differently.

## Introduction

Since spring of 2020, when the COVID-19 pandemic began to spread across the globe, universities in the US enforced lockdown orders on campus, such as transferring in-person classes to virtual classes, cancelling extracurricular activities, and executing various social distancing precautions throughout the fall semester of 2020 [1–7]. Heightened fears and worries of the unprecedented pandemic along with drastic disruption of routinized academic and social life, and financial and living situations negatively impacted college students’ quality of life [8–15]. During the fall semester of 2020, a majority of college students reported less physically active and higher rates of anxiety and depression impacted by the COVID-19 [4,5]. They felt unable to cope with increased worrying about their health and academic performance [5]. The increased mental burden and health concerns resulted in difficulties in concentrating, sleeping, and socializing [5].With the prevalence of loneliness and helplessness facing college students under the unusual circumstances [1–7], receiving strong social support is of paramount importance to mitigate the negative COVID-19 impact on their quality of life overall. There is an urgent need to explore how college students’ perceived social support is associated with impact of the COVID-19 on quality of life in order to provide insightful information for better coping with very challenging situations.

Perceived social support refers to individuals’ perceptions of receiving multidimensional social support sources and adequacy from family members, friends, and a significant other [15]. Perceived social support describes the degree to which individuals feel that their family members, friends, and significant others provide emotional, informational, instrumental, and appraisal supports for them during times of need [15–18]. Perceived social support acts as a buffer against stressful life events and adverse situations and serves as an important protector of physical and mental health [15–19]. In a nutshell, perceived social support plays an essential role in influencing and improving the quality of life across various populations [16–18]. Quality of life refers to individuals’ subjective perceptions of their physical, mental, psychosocial, and functional aspects of well-being in various contexts of life [7–9]. Quality of life involves individuals’ cognitive appraisal of and emotional reactions to their situations in life in relation to whether and/or the extent to which they achieve their expected goals, develop and maintain a healthy relationship with others, and feel satisfied with their living conditions [7–9,15]. Quality of life is a broad concept about the essence of health-related and multifaceted psychological well-being [7–9,15]. In short, quality of life is ‘an individual’s perception of their position in life in the context of the culture and value systems in which they live and in relation to their goals, expectations, standards and concerns’ [15,p.1403].

Previous studies have shown that perceived social support was positively associated with quality of life in cancer patients [20], older adults [21], and young adults [17,18]. The positive relationship between social support and quality of life is moderated by gender. For example, a study of 1930 cancer patients found that patients with lower perceived social support had significantly lower scores of global health-related quality of life, physical, emotional, cognitive, and social functioning and higher levels of depression [20]. In addition, the results indicated that male patients had a greater risk of lower perceived social support compared to female patients [20]. Similarly, in a study of an association of perceived social support with quality of life among 517 older adults aged 65 years and over, the results revealed that multidimensional perceived social support accounted for 22.1% of the total variance in quality of life [21]. Higher levels of multidimensional perceived social support were significantly associated with increased levels of quality of life [21]. Further, a study of 344 college students aged 17–26 showed that perceived social support explained 17% of the variance of depression [18]. Students with higher levels of perceived social support from family and friends had lower levels of depressive symptoms [18]. More importantly, the study found that higher levels of perceived social support acted as a protective buffer for depressive symptoms, even for college students facing a moderate level of stress [18]. However, inconsistent with the study results of gender differences in perceptions of social support [20], male college students had higher levels of perceived social support from family, friends, and a significant others compared to female students who had higher depression [18]. Likewise, a study of 315 first-year college students, who experienced a moderate level of perceived stress, reported higher levels of perceived social support were significantly associated with lower levels of perceived stress [17]. Despite female students having significantly higher levels of perceived stress than male students, a moderate negative relationship between perceived social support and perceived stress was observed for both female and male students [17].

Given the critical role that perceived social support plays as a determinant of quality of life in general, it is of great interest to understand how perceived social support influences the impact of COVID-19 on quality of life among college students during the fall semester of 2020, the most difficult and unpredictable time of everyone experienced over the past two years. The COVID-19 pandemic impacted college students’ quality of life in many ways during the fully shutdown fall semester of 2020. For example, a study showed 27% prevalence of loneliness among 1,964 college students [2]. One other study found an increase in alcohol consumption, especially among students with pre-existing depression and anxiety [10]. A study reported that 10% of interviewed college students aged 18–30 experienced moderate to severe levels of stress [7]. In addition, studies have shown that college students are highly susceptible to mental health issues [3]. However, to the best of our knowledge, there is a lack of studies examining the pressing and urgent issues among college students during the fall semester of 2020. Specifically, given barriers to socializing as a result of COVID-19, it is important to consider how changes in access to social support impacted the quality of life of college students.

Thus, the purpose of this study was three-fold: (1) to investigate the current status of perceived social support and the COVID-19 impact on quality of life in college students during the fall semester of 2022; (2) to examine the extent to which an overall perceived social support, and perceived social support from family members, friends, and a significant were associated with COVID-19 impact on quality of life among the total sample, and females and males, respectively; and 3) to further examine if there were differences in perceived social support between better and worse COVID-19 impact on quality of life groups among the total sample and by gender.

## Methods

### Participants and setting

Participants were 1259 students who were enrolled in a large public university in Midwest region of the US during the fall semester of 2020. The participants had a mean age of 21.53 (SD  =  3.470) years. 68% of the participants identified as female, 32% identified as male. Most of the participants (68%) were undergraduate students. Table 1 presents the demographic information of the participants in detail.

**Table 1.** Demographic information.

Variables	Frequency	Percentage
Gender		
Males	399	31.69
Females	860	68.30
Years of college study		
Freshman	277	22
Sophomore	189	15
Junior	214	17
Senior	176	14
Master’s students	214	17
Doctoral students	63	5
Professional degree	126	10
Ethnicity		
Hispanic	89	7
Non-Hispanic	1171	93
Race		
African American	38	3
Asian	277	22
Multiracial	113	9
White	806	64
Other	25	2

After obtaining approval of the study IRB application by the University of XXXX Institutional Review Board of Health and Behavioral Sciences (HUM00189120), we began to recruit participants using the university targeted email request system, the university learning management system named Canvas, and social media (Instagram). Students aged 17 and over and were enrolled in the university during the fall semester of 2020 were eligible for participation. The exclusion criteria was students who did not consent to participate. We sent an invitation letter via email to students (*n* = 2,000). The letter described the purpose of the study, the content and scope of questionnaires, the protocol for completing the study questionnaire including confidentiality and anonymity, time frame to complete it, and the opportunity to win a raffle of $25 Amazon gift cards. We also posted the invitation letter to Canvas, and an Instagram page.

### Data collection

The online questionnaire via Qualtrics, an online survey platform, was sent out during the first week of November 2020. Participants were given two weeks to anonymously complete two different questionnaires: the Multidimensional Scale of Perceived Social Support and the COVID-19 Impact on Quality of Life as well as demographic information: age, gender, education status, and race/ethnicity. Participants had to consent before proceeding with the questionnaire. The questionnaire was available via Qualtrics from 2 November to 16 November 2022. We received the first response on 2 November 2020, immediately we sent the Qulatrics questionnaire out on that day and obtained the last response on 16 November 2020. One week after the completion of the questionnaire, we sent thirty-one $25 Amazon gift cards to raffled participants who completed the questionnaire. During the time of this study conducted, a stay in place order for all university students was enforced halfway through of the semester.

#### The multidimensional scale of perceived social support (MSPSS)

The 12-item MSPSS consists of three sub-scales. Four items on each sub-scale are designed for individuals to self-rate their perceptions of receiving social support from significant others, friends, and family members with a 7-point rating scale (1 = very strongly disagree − 7 = very strongly agree) [15]. For example, a question assessing social support from significant others is: ‘There is a special person who is around when I am in need’. A question assessing social support from friends is: ‘I can talk about my problems to my friends’. An example of a family question is: ‘My family really tries to help me’. To calculate the score of each sub-scale, the average score of the questions regarding that sub-scale was taken. A total score was also calculated by taking the average score of all the questions. A higher score indicates a higher level of social support. The MSPSS has been found to be a very reliable scale for college students (*α* = 0.88) [15].

#### The COVID-19 impact on quality of life (COVID-19 QoL)

The questionnaire contains 6 questions designed for individual to self-rate the impact of COVID-19 on quality of life in physical and mental health as well as personal safety [22]. The participants responded to each question on a 5-point rating scale, (1 = completely disagree − 5 = completely agree). Examples of questions are ‘I think my physical health may deteriorate’, ‘I think my mental health has deteriorated’, and ‘I feel that my personal safety is at risk’. The total score was used by calculating the average score of each response to indicate a level of the COVID-19 QoL. Accordingly, the total score of the COVID-19 QoL ranged from 1 to 5, with 3 indicating neither agree nor disagree (neutral point). It is important to note that a lower score indicates a higher quality of life. In non-clinical samples, it is found to have a Cronbach alpha of 0.856 [19]. This is a valid questionnaire for the sample population.

### Sample size

We used G*Power 3.1.9.7 to calculate the study sample size with choosing *F* test for multiple regression analysis based on the study design, a moderate effect size (*f*^2^ = 0.15), 2-tailed an alpha-level of 0.01, a power of 95, and 4 predictors. The calculation showed the total sample size of the study requires 129 participants to attain 95% of power. Our sample size of 1296 participants highly exceeded the required sample size of 129.

### Data analysis

Of the 1633 total participants responding to our invitation email, 1296 consented to participate in the study. The response rate was 79%. Among the 1296 participants, 15 cases were identified as complete missing by means of a list-wise deletion methods and 22 were identified as outliers by using the SPSS_Explore with Tukey method. As the results of the data screening, a total of 37 cases were excluded from the final data analysis. 1259 participants’ responses comprised the final data set for conducting statistical analysis. Descriptive statistics of the study variables, including mean, standard deviation, skewness, and kurtosis, were calculated to conduct normality tests and to determine the participants’ current status of perceived social support from significant others, friends, and family, the overall perceived social support, and the COVID-19 QoL. The results skewness and kurtosis of the study variables ranged from −0.577 to −1.129, and from 0.152 to 1.956, respectively, indicating a normal distribution of each variable. Then, we conducted independent sample *t*-tests (Welch’s test was chosen due to unequal variance) to examine if there was any significant difference in each of the study variables between the male and female groups. To determine the extent to which perceived social support from significant others, friends, and family were associated with the COVID-19 QoL, we conducted multiple regression models for the total sample, the male group, and the female group. To further examine if there was a significant difference in the overall perceived social support between the two COVID-19 QoL groups. A score of 3, indicating ‘neither agree nor disagree’ on each of the six items on the COVID-19 QoL, was used to classify the participants into two COVID-19 QoL groups. The participants who scored ≥3 were in the worse COVID-19 QoL group, while the participants who scored  < 3 were in the better COVID-19 QoL group. ANCOVA was performed for the total sample while controlling for gender. Then, ANOVA was performed for the male group and the female group separately. A score of 3 on the COVID-19 QoL was used to classify the participants into two COVID-19 QoL groups. All statistical analyses were performed using IBM SPSS 27. A significance level of *p* < .05 was set for all statistical methods.

## Results

### Descriptive statistics

Table 1 presents demographic information for all participants, including gender, education year, ethnicity, and race. Table 2 presents the descriptive statistics of the study variables for the total sample and by gender. As shown in Table 2, for the total sample, the mean scores of perceived social support from significant others, friends, and family were 5.47, 5.53, and 5.30 on a scale of 1-7, respectively. For females, the mean scores of perceived social support from significant others, friends, and family were 5.58, 5.59, and 5.26, respectively. For males, the mean scores of perceived social support from significant others, friends, and family were 5.24, 5.40, and 5.39, respectively. A higher score on this scale indicated that the participants received a relatively high perceived social support from significant others, friends, and family. As seen in Table 3, the results of independent t-tests (Welch’s test) revealed that the female group scored significantly higher than the male group in the perceived social support from significant others (*t*  = −3.857, *df* = 686.81, *p* = .000), from friends (*t* = −2.48, *df* = 778.95, *p* = .013) and total perceived social support (*t* = −2.18, *df* = 702.74, *p* = .013). The results of independent *t*-tests (Welch’s tests) revealed no significant difference between the male and female groups with regards to family (*t* = 1.499, *df* = 771.59, *p* = .134).

**Table 2.** Descriptive statistics of the variables for the total sample and by gender.

	Total (*n* = 1259)	Male (*n* = 399)	Female (*n* = 860)
Variables	M (SD)	M (SD)	M (SD)
Age	21.53 (3.470)	21.61 (3.709)	21.52 (3.374)
PSS-significant others	5.47 (1.377)	5.24 (1.499)	5.58 (1.303)
PSS-friends	5.53 (1.269)	5.40 (1.262)	5.59 (1.282)
PSS-family	5.30 (1.403)	5.39 (1.408)	5.26 (1.399)
PSS total	5.45 (1.106)	5.22 (1244)	5.50 (1.070)
The COVID-19 QoL	2.35 (0.583)	2.219 (0.618)	2.41 (0.554)

**Table 3.** Independent samples test for variables between males and females.

Variable	*t*	*df*	*p*
PSS-significant others	−3.857	686.814	.000
PSS-family	1.499	771.591	.134
PSS-friends	−2.479	778.951	.013
PSS total	−2.184	702.737	.029
COVID-19 QoL	−5.542	704.816	.000

The total sample’s mean score of the COVID-19 QoL was 2.35. The maximum score attainable on this was 5, which indicates the lowest COVID-19 QoL. The mean score of the female group was slightly higher than that of the male group. Welch’s test indicated that the female group scored significantly higher than the male group in the COVID-19 QoL (*t* = −5.54, *df* = 704.82, *p* = .000), indicating that the female group had a worse COVID-19 QoL than the male group.

### Association of perceived social support with COVID-19 QoL

Table 4 presents the results of the multiple linear regression models with perceived social support form significant others, friends, and family predicting COVID-19 QoL for the total sample and by gender. For the total sample, the results showed that perceived social support from significant others, friends, and family significantly predicted COVID-19 QoL, accounting for 10% of the total variance when controlling for gender. Further, the results of the standard regression coefficients indicated that perceived social support from family (*β* = −0.237, *t* = −7.838, *p* = .000) was the only significant individual predictor of COVID-19 QoL. In contrast, perceived social support from significant others (*β* = −0.057, *t* = −1.522, *p* = .128) and friends (*β* = −0.013, *t* = −0.376, *p* = .707) were not significant individual predictors of COVID-19 QoL. The results indicated that a higher level of perceived social support from family is associated with a lower level of COVID-19 QoL.

**Table 4.** Multiple linear regression models with perceived social support form significant others, friends, and family predicting COVID-19 QoL for the total sample and by gender.

Variable	*R*	*R^2^*	*F*	*p*	*df*	*β*	*t*	*p*
Total sample								
Model	0.318	0.101	35.154	.000	(4,1254)			
Gender	0.158	5.826	0.000					
Significant others						−0.057	−1.522	.128
Friends						−0.013	−0.376	.707
Family						−0.237	−7.838	.000
Male group								
Model	0.350	0.123	18.407	.000	(3,395)			
Significant others						−0.071	−1.046	.296
Friends						0.048	0.751	.453
Family						−0.330	−5.901	.000
Female group								
Model	0.243	0.059	17.839	.000	(3,856)			
Significant others						−0.043	−0.960	.337
Friends						−0.041	−0.942	.347
Family						−0.201	−5.490	.000

Similarly, the results of the linear regression model indicated that the three sub-scales of the perceived social support significantly predicted COVID-19 QoL for the male and the female groups, explaining 12.3% of the total variance in COVID-19 QoL and 5.9% of the total variance respectively. Congruent with results from the total sample, for the female students, perceived social support from family (*β* = −0.201, *t* = −5.49, *p* = .000) was the only significant predictor of COVID-19 QoL. Perceived social support from significant others (*β* = −0.043, *t* = −0.960, *p* = .337) and friends (*β* = −0.041, *t* = −0.942, *p* = .347) were not significant individual predictors of the COVID-19 QoL. Similarly, the male students’ only significant predictor of COVID-19 QoL was perceived social support from family (*β* = −0.330, *t* = −5.901, *p* = .000). Perceived social support from significant others (*β* = −0.071, *t* = −1.046, *p* = .296) and friends (*β* = 0.048, *t* = −0.751, *p* = .453) were not significant individual predictors of COVID-19 QoL.

### Difference in perceived social support between better and worse quality of life groups

**Figure 1.**
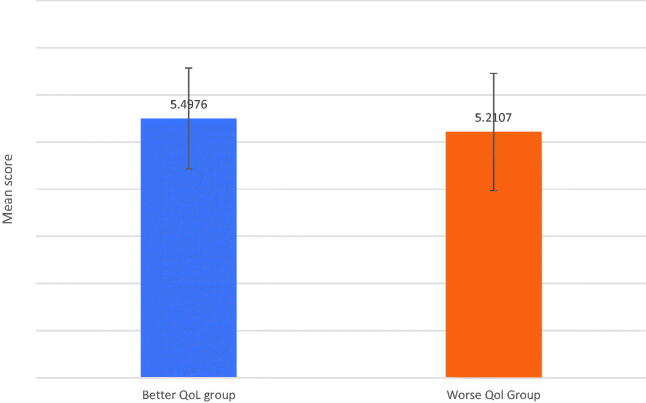
Mean scores of Total PSS between the Better QoL and the Worse QoL groups.

For the total sample, mean scores of the total perceived social support between worse COVID-19 QoL and better COVID-19 QoL groups are presented in Figure 1. The results of ANCOVA analysis with controlling for gender revealed significant group effects on the total perceived social support (*F* = 13.627, *p* = .000, *η^2^* = 0.011). The results indicate that the better COVID-19 QoL group scored significantly higher on the total perceived social support than the worse COVID-19 QoL group.

**Figure 2.**
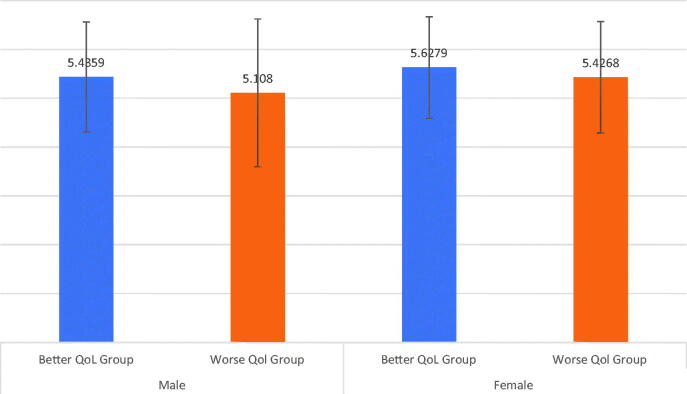
Mean scores of total PSS between the better QoL and worse QoL groups by sex.

Furthermore, the mean scores of the total perceived social support between the better COVID-19 QoL and the worse COVID-19 QoL groups stratified by gender were illustrated in Figure 2. The ANOVA results revealed a significant group effect on the total perceived social support for males (*F* = 10.054, *p* = .002, *η^2^* = 0.025) and females (*F* = 5.978, *p* = .015, *η^2^* = 0.007). These results indicated that the male better COVID-19 QoL group scored significantly higher on the total perceived social support compared to the male worse COVID-19 QoL group. Similarly, the female better COVID-19 QoL group scored significantly higher on the total perceived social support compared to the female worse COVID-19 QoL group.

## Discussion

This study was central to investigating the current status of perceived social support and of the COVID-19 impact on QoL, associations of the perceived social support with the COVID-19 QoL, and differences in perceived social support between the better COVID-19 QoL group and the worse COVID-19 QoL group for the total sample and by gender. It is important to note that the study was conducted during the first year of the COVID-19 pandemic and throughout the first fully-lockdown semester due to the pandemic. Even in this unprecedented situation, the participating college students reported a moderately high level of perceived social support from significant others, friends, and family and a relatively low level of the COVID-19 impact on quality of life. Further, female students had a significantly higher level of perceived social support from significant others, friends, and total perceived social support than male students. In contrast, female students had a greater negative impact of COVID-19 on their quality of life compared to their male counterparts.

The unexpected result of our study, students having less negative impact of the COVID-19 on their quality of life, were inconsistent with findings reported in other studies [23,24]. Hansel et al. [23] reported that COVID-19 experiences and fear of developing the infection likely exacerbated existing mental health problems and increased anxiety and depressive symptoms among young adults. Mental health condition is predictive of quality of life [23]. Moreover, young adults who experienced the COVID-19 infection reported lower quality of life [23]. One possible reason for the participating students’ quality of life which was less negatively impacted by COVID-19 pandemic might be related to education levels. Of 1259 participants in this study, 68% were undergraduate students, 22% were graduate students, and 10% were pursuing a professional degree. A study showed that education levels were positively associated with taking responsibility for coping with health-related issues and quality of life during the pandemic [24]. The participating students’ socioeconomic status and racial and ethnic groups might be another combined explanatory factor for having a less negative impact of COVID-19 on their quality of life. People with low socioeconomic status and those of certain racial and ethnic groups, including African American, Hispanic, and Native American, have disproportionately experienced low quality of life during the pandemic [25]. In this study, only 7% of the participating students were Hispanic, compared to 93% non-Hispanic; African Americans only accounted for 3% of the total sample. Although our study did not include socioeconomic status in the data collection, the university itself is a highly academically competitive university with a high tuition fee, especially the out of state tuition fee being as high as Ivy League universities. Accordingly, a small portion of the student body may from low-income families. The absence of chronic health conditions might another factor explaining why our participating students were less negatively impacted by the COVID-19 pandemic. People with chronic health conditions, such as heart disease, diabetes, cancer, chronic obstructive pulmonary disease, chronic kidney disease, and obesity, are most vulnerable to the complications of COVID-19 and at high risk for severe illness from COVID-19 [25]. Fortunately, most of college students are in good health condition and they are known to be the least vulnerable population for severe illness from the pandemic [25]. More importantly, the current study found that participating students’ relatively high level of perceived social support overall was a significant contributor to their experiencing less negative impact of the COVID-19 on their quality of life amid the severe outbreak of the pandemic.

Regarding the relationship of perceived social support from three different resources, significant others, friends, and family, with the COVID-19 QoL, our results indicated that a higher level of perceived social support from family was the only significant individual contributor to a better level of the COVID-19 QoL among the total sample. Similarly, when examining the association by gender, a higher level of perceived social support from family was significantly associated with a better level of COVID-19 QoL for both males and females. Furthermore, the participants, regardless of males and females groups, who had a higher level of perceived social support overall had a better level of COVID-19 QoL. However, perceived social support from significant others and friends were not significantly associated with COVID-19 QoL for the total sample, and for both females and males, respectively during the first semester fully impacted by the pandemic in relation to the campus shutdown. Another study examining the mental health of young adults in the US had a similar finding that social support from family, but not from significant others or friends, was associated with lower rates of depression during the COVID-19 pandemic [13]. Although further examination is required to understand how different social relationships interact with the impact of COVID-19 on quality of life, these results suggest that family support may play a unique role in improving health-related quality of life for college students throughout the pandemic.

However, it is noticeable that the better quality of life group reported significantly higher overall perceived social support for the total sample as well as for males and females. These results indicate that having a social network to rely on may act as a buffer during stressful times such as the COVID-19 pandemic. This is congruent with a study demonstrating the benefits of social support in mediating emotional states such as anxiety during the COVID-19 [14]. An important question is whether virtual interactions have the potential to serve the same social support as face-to-face socialization. Previous research examining the differences between in-person and online support indicates that virtual social support may reduce stress response as effectively as face-to-face, as long as the virtual interaction is with another person [26]. These findings suggest that maintaining social interaction *via* any platform available may attenuate stressors inflicted by COVID-19 and buffer the experience of social distancing. Overall, the importance of social support as related to the impact of COVID-19 on the quality of life of young adults amid the pandemic is clear.

Our findings on gender differences point out how males and females may experience perceived social support and quality of life differently during the pandemic. Females scored significantly higher on total perceived social support, social support from significant others and from friends, yet al.so reported a worse quality of life during COVID-19, compared to their male counterparts. The reasons for these differences are unclear, but a worse quality of life during COVID-19 in females suggests that female college students were impacted more negatively by the pandemic. A similar study investigating gender differences in responses to COVID-19 in China found that females experienced higher psychological stress during this time compared to males [27]. With respect to the observed difference in perceived social support, it is possible that female college students used their social network to a higher degree than males as a result of experiencing a relatively worse quality of life. However, no causal conclusions can be made. Further investigation is needed to understand these complex relationships.

To the best of our knowledge, no other studies have looked into gender differences in the relationship between perceived social support and the COVID-19 impact on quality of life. However, our findings contradict the results of a study [28], in which perceived social support was higher in males than females with depression. Another study showed that the relationship between stress, perceived social support, and resilience was stronger in females compared to males [29]. Other research argues there is a gap in understanding the relationship between social support and health, as females and minorities are often underrepresented in these studies [30]. Future research should investigate specifically how gender plays a role in perceived social support, and whether changes in social accessibility during COVID-19 have had different effects on males and females. Future research should also investigate the effects of remote socialization as well as online learning in higher education in relation to perceived social support, and whether there are gender differences in the impacts of virtual interaction.

There are several limitations of this study to note. First, related to the voluntary and self-report nature of this study, students volunteered to take the study survey. Interestingly, nearly 70% of the sample population identified as female. This imbalance in gender representation may have biased our results, particularly those pertaining to gender differences. Second, given the cross-sectional study design, we did not collect baseline data before the pandemic in order to quantify changes. We relied on self-report data to examine changes in quality of life impacted by COVID-19 and how perceived social support is associated with it. Thus, we are unable to say that COVID-19 played a causal role in our findings. Third, we did not collect some potentially important covariates, such as socioeconomic background, COVID-19 infection, chronic health condition, and students from different schools/colleges with different majors, which may play a role in perceived social support and quality of life during the COVID-19 pandemic. Future studies should include these factors in data collection and examine them as covariates within study outcomes.

## Conclusion

In conclusion, participating students in this study reported a moderately higher level of perceived social support from significant others, friends, and family, and overall perceived social support. The negative impact of the COVID-19 on quality of life was modest among the total sample. However, females reported higher perceived social support on all sub-scales except family, but experienced a relatively worse COVID-19 QoL, compared to males. Second, although perceived social supports from significant others, friends, and family were collectively significant contributors to COVID-19 QoL, perceived social support from family was the only stand-alone significant predictor of COVID-19 QoL for the total sample and for the females and the males groups. Finally, participants with higher perceived social support had better COVID-19 QoL regardless of females and males. This study suggests that multidimensional social support may buffer the negative impact of COVID-19 on quality of life during very challenging and unpredictable times. Moreover, perceived social support from family plays the most essential role in determining the impact of COVID-19 on quality of life. Our results also suggest that the pandemic may have impacted the quality of life of males and females differently. However, further research is needed to understand the roots of these differences. Overall, this study helps inform families, universities, and institutions to support their students throughout these uncertain times.

## Data Availability

The data that support the findings of this study are available on request from the corresponding author. The data are not publicly available due to them containing information that could compromise the privacy of research participants.
